# Health‐Related Quality of Life and Psychological Burden of Patients With Vitiligo in Japan

**DOI:** 10.1111/1346-8138.70059

**Published:** 2025-11-27

**Authors:** Naoki Oiso, Yasutaka Mizoro, Kazumasa Kamei, Kenichi Yamanaka, Masato Hoshi, Kouki Nakamura

**Affiliations:** ^1^ Department of Dermatology Kindai University Nara Hospital Nara Japan; ^2^ Specialty Care Medical Affairs Pfizer Japan Inc. Tokyo Japan; ^3^ Japan Access and Value Pfizer Japan Inc. Tokyo Japan

**Keywords:** observational study, patient reported outcome measures, psychosocial functioning, quality of life, vitiligo

## Abstract

Vitiligo is a disorder characterized by depigmentation of the skin and is known to impact patients' health‐related quality of life (HRQoL). In Japan, HRQoL studies on vitiligo remain limited in size and scope, and factors contributing to impaired HRQoL, as well as the psychological burden, have not been adequately evaluated. This study aimed to assess HRQoL and symptoms of anxiety and depression in patients with vitiligo in Japan. A web‐based survey was completed by 271 patients with vitiligo aged 18 to 79 years. The survey included the 12‐item Short Form Health Survey version 2 (SF‐12v2) and the Dermatology Life Quality Index (DLQI) to assess HRQoL, as well as the Hospital Anxiety and Depression Scale (HADS). The role/social component summary score (mean ± standard deviation) of the SF‐12v2 in vitiligo patients was 45.8 ± 14.5, which was lower than the Japanese population norm (national standard values for Japanese). Based on the DLQI, which is specific to dermatologic diseases, 62.7% of patients reported at least a small effect on their daily lives. HADS anxiety and depression scores showed that 38.0% and 40.3% of patients, respectively, were classified as doubtful or definite cases. Subgroup analyses revealed that higher DLQI and HADS scores were associated with sex, age, disease duration, affected body surface area (BSA), and history of relapse. Notably, nonlinear associations in these measures were observed for disease duration and affected BSA, with the highest scores seen in patients with an intermediate range of disease duration and affected BSA. These findings underscore the need for timely and sustained treatment strategies to control symptoms, reduce psychological distress, and prevent relapse, which may help not only improve clinical outcomes but also maintain long‐term HRQoL in individuals with vitiligo.

## 1 Introduction

Vitiligo is an autoimmune disease characterized by depigmentation caused by loss of melanocytes [1]. It is estimated that at least 1% of the global population is affected by vitiligo, with a lifetime prevalence ranging from 0.4% to 2% [2, 3, 4]. There is no difference in incidence between sexes, and the disease can develop at any age [1]. According to a population‐based, multinational study conducted in Europe, Japan, and the United States, the prevalence of vitiligo in these countries was 1.6%, 0.5%, and 1.4%, respectively [5].

Vitiligo is known to impact health‐related quality of life (HRQoL) [1, 5, 6, 7], and in particular, there are reports of increased psychosocial burden [1, 5, 7, 8, 9]. Factors reported to affect HRQoL include sex, age, skin type, body surface area (BSA) of depigmentation, and disease progression [5, 9, 10, 11]. In addition, vitiligo is often comorbid with other dermatologic diseases such as alopecia areata and atopic dermatitis [1, 8, 12] and has also been associated with anxiety and depression [1, 8, 9]. There is a clinical consensus that psychological support is essential in the management of vitiligo [6, 13, 14]. Therefore, evaluating HRQoL is an important part of patient care.

The impact of vitiligo on HRQoL varies by skin type, ethnicity, and geographic region [10], suggesting the need for large‐scale, country‐ or region‐specific analyses. However, previous studies evaluating HRQoL in patients with vitiligo in Japan have been limited in sample size and scope and have not assessed the demographic or disease‐related factors that are associated with impaired HRQoL [5, 15]. Furthermore, to the best of our knowledge, no study to date has specifically assessed the psychological impact of vitiligo by solely focusing on patients in Japan.

This study used a web‐based survey to assess HRQoL, anxiety, and depression in patients with vitiligo in Japan. The primary objective was to identify which domains of HRQoL are more affected in patients with vitiligo compared with the Japanese population norms (national standard values for Japanese). The secondary objectives were to assess the impact of vitiligo on daily life using a dermatology‐specific measure and to evaluate psychological burden using indices of anxiety and depression. These outcomes were analyzed in relation to patient demographic variables such as sex and age and clinical variables such as disease duration, severity, and history of relapse, in order to explore the factors associated with impaired HRQoL and psychological burden.

## 2 Methods

### 2.1 Study Design and Participants

This study was a web‐based survey conducted between October and November 2024 in patients with vitiligo. Participants were recruited from a survey panel maintained by Macromill Carenet Inc. (Tokyo, Japan). A screening survey was first conducted to assess eligibility among those who provided consent. Participants deemed eligible by the screening proceeded to complete the web‐based survey form.

Patients were eligible if they reported being diagnosed with vitiligo by a doctor (not limited to dermatologists), were between 18 and 79 years of age, were able to access and respond to the web‐based survey form, and consented to participate after receiving a sufficient explanation of the study objectives and content. To minimize confounding effects of other diseases on HRQoL, patients who self‐reported a diagnosis of at least one designated intractable disease (i.e., a rare disease with an unclear cause, no established treatment or diagnostic criteria, and the need for long‐term care, as established by the Japanese Ministry of Health, Labour and Welfare) were excluded from the study.

### 2.2 Endpoints

The primary endpoint was the comparison of scores on the 12‐item Short Form Health Survey version 2 (SF‐12v2) [16, 17]. The analysis included eight subscale scores—physical functioning (PF), role physical (RP), bodily pain (BP), general health (GH), vitality (VT), social functioning (SF), role emotional (RE), and mental health (MH)—and three component summary scores—physical component summary (PCS), mental component summary (MCS), and role/social component summary (RCS). The scores for the SF‐12v2 were interpreted using norm‐based scoring (NBS), which considers the deviation in scores from the Japanese population norms (national standard values for Japanese). The Japanese population norms used in this study were the average values from a survey conducted in 2017 [18].

Other endpoints included the Dermatology Life Quality Index (DLQI) score [19, 20], and the Hospital Anxiety and Depression Scale (HADS) scores for anxiety (HADS‐A) and depression (HADS‐D) [21, 22, 23, 24]. The DLQI ranges from 0 to 30, categorizing the impact of dermatological disease on daily life as follows: 0–1, no effect at all; 2–5, small effect; 6–10, moderate effect; 11–20, very large effect; and 21–30, extremely large effect [19, 20]. HADS‐A and HADS‐D are each scored from 0 to 21, with 0–7 indicating a non‐case, 8–10 indicating a doubtful case, and 11–21 indicating a definite case [21, 22, 23, 24].

Collected patient background and self‐reported clinical variables are shown in Table 1. Disease severity was rated from 0 (no symptoms) to 10 (most severe) [10]. Affected BSA was estimated by participants using the “hand unit” method, in which one hand unit corresponds to approximately 1% of total BSA [25]. SF‐12v2, DLQI, HADS‐A, and HADS‐D scores were analyzed by subgroup according to disease duration and affected BSA, and multivariate analyses were performed using background variables as independent variables. As an exploratory analysis, correlations among SF‐12v2, DLQI, HADS‐A, and HADS‐D scores were also assessed.

**TABLE 1:** Participant characteristics.

*n*	271
Age (years), mean ± SD	45.1 ± 15.8
Sex, *n* (%)	
Female	138 (50.9)
Comorbidities, *n* (%)^a^	
Yes	168 (62.0)
Hypertension	48 (17.7)
Atopic dermatitis	40 (14.8)
Dyslipidemia	26 (9.6)
Cataract	23 (8.5)
Depression	21 (7.7)
Fitzpatrick skin type, *n* (%)^b^	
Type I	23 (8.5)
Type II	87 (32.1)
Type III	94 (34.7)
Type IV	34 (12.5)
Type V	20 (7.4)
Type VI	9 (3.3)
Do not want to answer	4 (1.5)
Disease duration, *n* (%)	
≤ 6 months	8 (3.0)
> 6 months to ≤ 1 year	14 (5.2)
> 1 year to ≤ 3 years	46 (17.0)
> 3 years to ≤ 5 years	30 (11.1)
> 5 years to ≤ 10 years	53 (19.6)
> 10 years	103 (38.0)
Unknown	17 (6.3)
Current affected sites, *n* (%) (multiple responses allowed)	
Face	104 (38.4)
Neck	75 (27.7)
Abdomen	63 (23.2)
Arms (shoulders to wrists)	57 (21.0)
Hands (distal to wrists)	74 (27.3)
Legs (groin to ankles)	62 (22.9)
Affected BSA, *n* (%)^c^	
< 1%	110 (44.9)
≥ 1% to < 4%	67 (27.4)
≥ 4% to < 10%	40 (16.3)
≥ 10% to < 25%	15 (6.1)
≥ 25% to < 50%	6 (2.5)
≥ 50%	7 (2.9)
History of relapse, *n* (%)	
Symptoms have never improved	110 (40.6)
Symptoms improved and then relapsed	86 (31.7)
Symptoms improved without relapsing	75 (27.7)
Current treatment for vitiligo, *n* (%)	
Yes	89 (32.8)
Unknown	28 (10.3)
None	154 (56.8)
Family history of vitiligo	
Yes	33 (12.2)

Abbreviations: BSA, body surface area; SD, standard deviation.

^a^ Only the top five comorbidities are shown.

^b^ S. Eilers, D. Q. Bach, R. Gaber, H. Blatt, Y. Guevara, K. Nitsche et al., “Accuracy of Self‐Report in Assessing Fitzpatrick Skin Phototypes I through VI,” *JAMA Dermatol* 149, no. 11 (2013): 1289–1294, doi: 10.1001/jamadermatol.2013.6101; and T. B. Fitzpatrick, “Soleil et Peau,” *J Med Esthet* 2, (1975): 33–34.

^c^ Data on affected BSA are available for 245 participants (26 participants who answered as having no lesions are excluded).

### 2.3 Statistical Analysis

Descriptive statistics are presented as mean ± standard deviation (SD) for continuous variables and as *n* (%) for categorical variables. SF‐12v2, DLQI, HADS‐A, and HADS‐D scores were calculated across categories of disease duration and affected BSA, and trends were assessed using linear and quadratic contrasts in analysis of variance. For disease duration, weights for the linear contrast were set at equal intervals from −3 to +3, ranging from “≤ 6 months” to “> 10 years”. For affected BSA, weights for the linear contrast were set at equal intervals from −3 to +3, ranging from “no lesions” to “≥ 50% of BSA”.

Linear regression models with forced entry were applied to examine associations between demographic or patient background characteristics as independent variables and HRQoL or psychological outcomes, including SF‐12v2, DLQI, HADS‐A, and HADS‐D scores, as dependent variables. Spearman's rank correlation coefficient (ρ) was used to assess correlations among the SF‐12v2 component summary scores, DLQI, HADS‐A, and HADS‐D scores. Two‐sided *p*‐values were calculated to assess statistical significance.

## 3 Results

### 3.1 Patient Characteristics

Among 250 812 individuals in the survey panel, 271 met the eligibility criteria and completed the survey form; all these participants were included in the analysis. The mean age of participants was 45.1 years, with an approximately equal proportion of male and female patients (Table 1). Comorbidities other than vitiligo that participants reported being diagnosed with included hypertension (17.7%), atopic dermatitis (14.8%), dyslipidemia (9.6%), cataract (8.5%), and depression (7.7%). Regarding Fitzpatrick skin type, 75.3% were classified as type I–III (light skin). A total of 57.6% had been diagnosed with vitiligo for over 5 years. The most common body site affected by depigmentation was the face (38.4%), followed by the hands (27.3%). A total of 27.8% had depigmented areas accounting for 4% or more of their BSA. Relapse after symptom improvement was reported by 31.7%. Among participants, 32.8% reported they were currently receiving treatment for vitiligo.

### 3.2 SF‐12v2 Results

**FIGURE 1:**
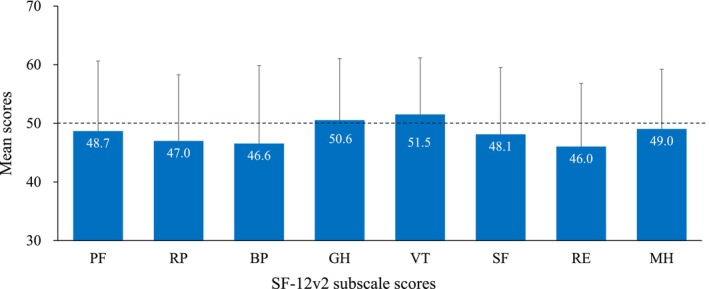
Comparison of SF‐12v2 subscale scores with the Japanese population norms (score = 50). Data are shown as mean ± standard deviation. BP, bodily pain; GH, general health; MH, mental health; PF, physical functioning; RE, role emotional; RP, role physical; SF, social functioning; SF‐12v2, 12‐item Short Form Health Survey version 2; VT, vitality.

**FIGURE 2:**
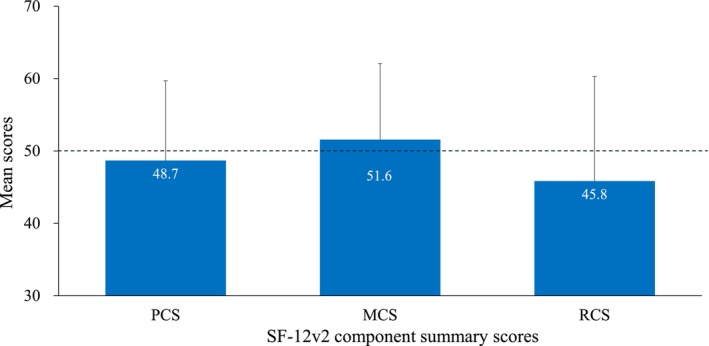
Comparison of SF‐12v2 component summary scores with the Japanese population norms (score = 50). Data are shown as mean ± standard deviation. MCS, mental component summary; PCS, physical component summary; RCS, role/social component summary; SF‐12v2, 12‐item Short Form Health Survey version 2.

Compared with the Japanese population norms, the norm‐based scores on four SF‐12v2 subscales—RP, BP, SF, and RE—were lower in the patients of this study, with RE being the lowest (Figure 1). Non‐transformed scores (0–100) for each subscale are presented in Figure S1. Among the norm‐based SF‐12v2 component summary scores, the mean RCS score was notably lower than the Japanese population norm, with a mean ± SD of 45.8 ± 14.5 (Figure 2).

### 3.3 DLQI/HADS Results

Based on the DLQI categorical classification for the effect on a patient's life, the proportions of patients in each category were as follows: small effect (28.8%), moderate effect (15.5%), very large effect (14.8%), and extremely large effect (3.7%) (Table 2). The DLQI score (mean ± SD) was 5.3 ± 6.3. For HADS‐A, the proportions of patients in each category were as follows: non‐case (62.0%), doubtful (19.2%), and definite (18.8%). The HADS‐A score (mean ± SD) was 5.9 ± 4.4. For HADS‐D, patient proportions were as follows: non‐case (59.8%), doubtful (20.7%), and definite (19.6%). The HADS‐D score (mean ± SD) was 5.8 ± 4.4.

**TABLE 2:** DLQI and HADS scores.

DLQI total score	
Mean ± SD	5.3 ± 6.3
DLQI class, *n* (%)	
No effect at all: 0–1	101 (37.3)
Small effect: 2–5	78 (28.8)
Moderate effect: 6–10	42 (15.5)
Very large effect: 11–20	40 (14.8)
Extremely large effect: 21–30	10 (3.7)
HADS‐A score	
mean ± SD	5.9 ± 4.4
HADS‐A class, *n* (%)	
Non‐case: 0–7	168 (62.0)
Doubtful: 8–10	52 (19.2)
Definite: 11–21	51 (18.8)
HADS‐D score	
mean ± SD	5.8 ± 4.4
HADS‐D class, *n* (%)	
Non‐case: 0–7	162 (59.8)
Doubtful: 8–10	56 (20.7)
Definite: 11–21	53 (19.6)

Abbreviations: DLQI, Dermatology Life Quality Index; HADS‐A, Hospital Anxiety and Depression Scale‐anxiety; HADS‐D, Hospital Anxiety and Depression Scale‐depression; SD, standard deviation.

### 3.4 Correlations Among Outcome Measures

Among the three SF‐12v2 component summary scores, the RCS score showed the strongest correlations with DLQI (*ρ* = −0.409, *p* < 0.001), HADS‐A (*ρ* = −0.535, *p* < 0.001), and HADS‐D (*ρ* = −0.449, *p* < 0.001) (Table S1). For the PCS score, these correlation coefficients were *ρ* = −0.321 (*p* < 0.001), *ρ* = −0.194 (*p* = 0.001), and *ρ* = −0.231 (*p* < 0.001), respectively. For the MCS score, they were *ρ* = −0.072 (*p* = 0.235), *ρ* = −0.300 (*p* < 0.001), and *ρ* = −0.356 (*p* < 0.001), respectively. Lastly, the DLQI score was correlated with both HADS‐A (*ρ* = 0.605, *p* < 0.001) and HADS‐D (*ρ* = 0.562, *p* < 0.001).

### 3.5 Vitiligo‐Related Factors Associated With Outcomes

**FIGURE 3:**
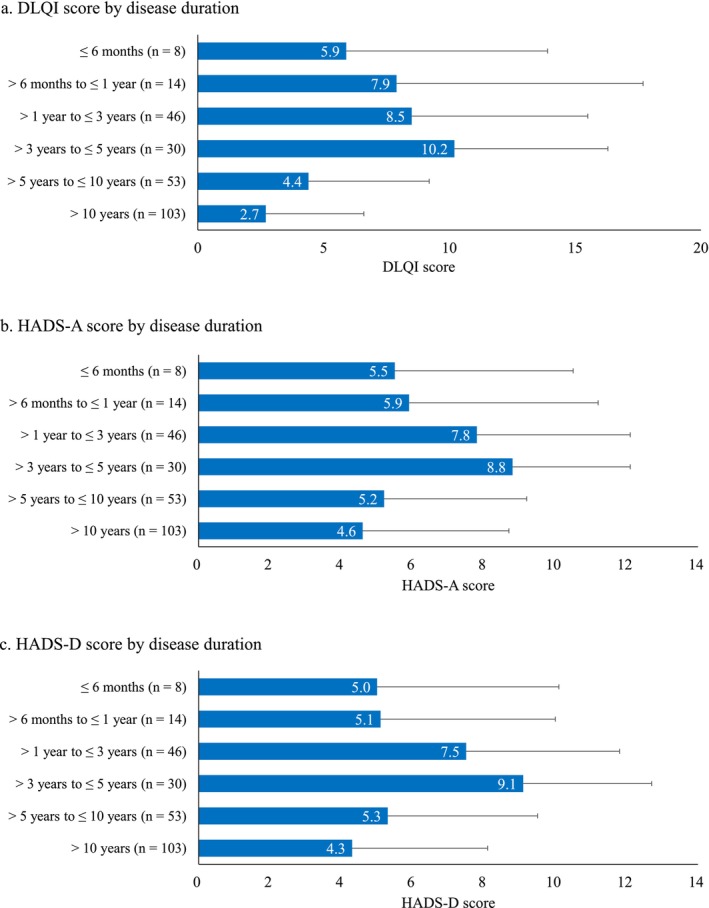
DLQI (a), HADS‐A (b) and HADS‐D (c) scores by disease duration. DLQI, Dermatology Life Quality Index; HADS‐A, Hospital Anxiety and Depression Scale‐anxiety; HADS‐D, Hospital Anxiety and Depression Scale‐depression.

**FIGURE 4:**
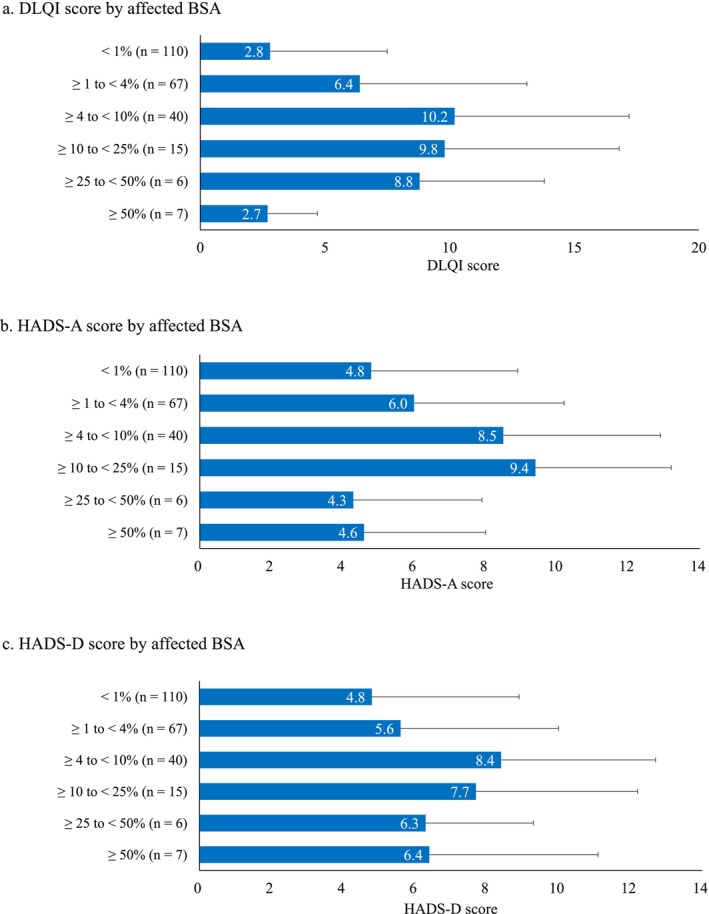
DLQI (a), HADS‐A (b) and HADS‐D (c) scores by affected BSA. BSA, body surface area; DLQI, Dermatology Life Quality Index; HADS‐A, Hospital Anxiety and Depression Scale‐anxiety; HADS‐D, Hospital Anxiety and Depression Scale‐depression.

We examined the associations between vitiligo‐related factors (disease duration and affected BSA) and HRQoL, anxiety, and depression. For disease duration, the highest mean ± SD scores for DLQI, HADS‐A, and HADS‐D were observed in patients with a disease duration of > 3 to ≤ 5 years (10.2 ± 6.1, 8.8 ± 3.3, and 9.1 ± 3.6, respectively) (Figure 3). The RCS score of the SF‐12v2 was lowest (mean: 39.1) in patients with a disease duration of > 6 months to ≤ 1 year (Figure S2). No apparent trends were observed for the MCS and PCS scores across disease duration groups. Regarding affected BSA, the highest mean ± SD scores for DLQI and HADS‐D were observed in patients with an affected BSA of ≥ 4 to < 10% (10.2 ± 7.0 and 8.4 ± 4.3, respectively). For HADS‐A, the highest mean ± SD score was observed in patients with an affected BSA of ≥ 10 to < 25% (9.4 ± 3.8) (Figure 4). For SF‐12v2 component summary scores, the mean ± SD score for PCS was the lowest in patients with an affected BSA of ≥ 10 to < 25% (39.6 ± 17.5), and the mean ± SD score for RCS was lowest in patients with an affected BSA of ≥ 4 to < 10% (40.5 ± 16.6) (Figure S3).

Because no linear trends of scores were observed in relation to either disease duration or affected BSA, we performed nonlinear trend tests. Quadratic contrast estimates showed bell‐shaped associations for disease duration with DLQI (−4.803, *p* < 0.001), HADS‐A (−2.943, *p* = 0.002), and HADS‐D (−3.258, *p* < 0.001) (Table S2). Similarly, affected BSA showed bell‐shaped associations for DLQI (−6.544, *p* < 0.001) and HADS‐A (−3.408, *p* = 0.002) (Table S2). A cup‐shaped association was also observed between affected BSA and SF‐12v2 RCS score (8.143, *p* = 0.029).

### 3.6 Multivariate Analysis

Multivariate analysis identified the following factors associated with a lower SF‐12v2 RCS score: younger age, disease duration < 5 years, affected BSA of ≥ 4% to < 10%, and family history of vitiligo (Table 3). A higher DLQI score was also associated with several factors, including affected BSA, history of relapse, family history of vitiligo, sex, age, disease duration, and affected body site. For HADS‐A, factors associated with a higher score were affected BSA, history of relapse, age, disease duration, and current treatment. Lastly, a higher HADS‐D score was associated with history of relapse, age, disease duration, and current treatment. In the regression models used to evaluate the influence of patient background on each outcome measure, the adjusted coefficient of determination (*R* ^2^) was highest for DLQI (*R* ^2^ = 0.504), followed by HADS‐A (*R* ^2^ = 0.308) and HADS‐D (*R* ^2^ = 0.264).

**TABLE 3:** Factors associated with HRQoL.

	SF‐12v2 PCS	SF‐12v2 MCS	SF‐12v2 RCS	DLQI	HADS‐A	HADS‐D
	Coefficient	Coefficient	Coefficient	Coefficient	Coefficient	Coefficient
Sex (female vs. male)	−2.412	−2.678	2.631	−2.263***	−0.522	−0.816
Age (per year)	−0.152***	0.000	0.251***	−0.077***	−0.063***	−0.049**
Comorbidities (yes vs. none)	−1.918	1.600	0.594	−0.518	−0.074	−0.875
Fitzpatrick skin type (dark skin [IV–VI] vs. I–III)	2.072	0.147	−0.696	−0.090	−0.308	−0.307
Disease duration (> 5 years or unknown vs. ≤ 5 years)	3.784**	−0.644	**4.463**	−3.427***	−1.721***	−1.698**
Affected site (face and/or hands vs. none)	2.787*	−2.112	3.157	−1.917**	−0.408	−0.841
Affected BSA (vs. no lesions)						
< 1%	7.837**	2.795	−5.586	−0.034	−0.497	−0.626
≥ 1% to < 4%	3.491	4.760	−5.324	1.693	−0.509	−1.159
≥ 4% to < 10%	−1.766	4.803	**−8.158**	4.821***	1.265	0.728
≥ 10% to < 25%	−6.435	0.651	−8.600	5.470**	3.230*	1.298
≥ 25% to < 50%	1.081	4.208	−9.812	7.977***	−0.440	1.292
≥ 50%	−5.234	4.106	−7.969	1.682	−0.251	0.844
Current severity (per 1‐point increase)	0.285	−0.380	−0.175	0.067	0.045	**0.226**
History of relapse (vs. no experience)						
Symptoms have never improved	−1.084	−1.766	−0.483	1.050	**1.424**	0.464
Symptoms improved and then relapsed	−3.031	−3.398	−2.842	3.065***	2.882***	2.301**
Currently receiving treatment for vitiligo (yes vs. no)	−1.731	**5.955**	−0.865	1.064	−2.606**	−3.008***
Family history of vitiligo (yes vs. no/prefer not to say)	−0.838	**3.995**	−6.969**	3.704***	0.703	−0.410
Adjusted coefficient of determination (*R* ^2^)	0.187	0.027	0.182	0.504	0.308	0.264

*Note:* The bold values indicating *p* < 0.05. * *p* < 0.05, ** *p* < 0.01, *** *p* < 0.001.

Abbreviations: BSA, body surface area; DLQI, Dermatology Life Quality Index; HADS‐A, Hospital Anxiety and Depression Scale‐anxiety; HADS‐D, Hospital Anxiety and Depression Scale‐depression; HRQoL, health‐related quality of life; MCS, mental component summary; PCS, physical component summary; RCS, role/social component summary; SF‐12v2, 12‐item Short Form Health Survey version 2.

## 4 Discussion

In this study, we evaluated the impact of vitiligo on HRQoL and psychological burden in patients. Compared with the Japanese population norm, the SF‐12v2 RCS score was lower in patients with vitiligo, indicating that vitiligo affects role and social functioning. Additionally, the SF‐12v2 subscale scores of RP, BP, SF, and RE were lower in patients with vitiligo than the Japanese population norms, with RE showing the lowest score. RE reflects restrictions in daily role activities due to emotional reasons; thus, our findings suggest that vitiligo affects psychosocial functioning.

The DLQI is a dermatology‐specific measure [19, 20] and has been widely used for HRQoL assessment in patients with vitiligo [10]. In this study, 62.7% of patients reported at least a small effect on their daily lives, indicating that patients with vitiligo experience a substantial burden in their daily lives. The mean DLQI score in this study was 5.3, which is similar to the findings of previous studies involving patients in Japan [15]. Moreover, this score is consistent with previous studies conducted in China and South Korea, countries with racial and regional proximity to Japan [10].

Vitiligo has been shown to impose a significant psychological burden on patients [7, 11, 13, 26], with previous studies outside of Japan using the HADS demonstrating associations with both anxiety and depression [14, 27, 28]. In the present study, the proportions of patients classified as definite were 18.8% for HADS‐A and 19.6% for HADS‐D, highlighting the need to consider the psychological burden associated with the disease. However, a Japanese claims database study reported that only 2.6% and 1.8% of patients with vitiligo had received diagnoses of anxiety and depression, respectively [8], suggesting that many patients with vitiligo in Japan may have undiagnosed psychological distress.

In the multivariate analysis, the RCS, DLQI, or HADS scores were associated with sex, age, disease duration, affected body site, affected BSA, history of relapse, and family history. These associations are consistent with previous studies on HRQoL in patients with vitiligo [5, 10]. While some of these studies were conducted in Japan, they did not utilize standardized instruments such as the DLQI or HADS to assess HRQoL [5, 15]. In this study, HRQoL impairment assessed by the RCS and DLQI scores was associated with younger age, shorter disease duration (≤ 5 years), and family history. Younger age and shorter disease duration were also associated with increased tendencies toward anxiety and depression measured by the HADS. Female patients had better HRQoL measured by DLQI than male patients, which contradicts previous reports indicating worse HRQoL among female patients [9, 10]. This discrepancy may be due to differences in background characteristics between the male and female patients or to factors other than sex. This study also found worse HRQoL and a higher tendency toward anxiety and depression in patients who had experienced relapse, which aligns with guidance from the International e‐Delphi Consensus [29] that emphasizes the importance of continued treatment in vitiligo to maintain repigmentation and disease control.

We analyzed the associations between DLQI and HADS scores and either disease duration or affected BSA, and identified a bell‐shaped trend, with the greatest psychological burden observed in patients with moderate disease duration or BSA involvement. Similar patterns have been reported in previous studies of vitiligo and other skin diseases. For example, one study found that patients with longer vitiligo duration (> 10 years) had better HRQoL than those with shorter duration [5], and another study found that patients with ≤ 5 or 15–20 years of disease duration had better HRQoL than those in the intermediate range, although the difference was not statistically significant [30]. Additionally, in alopecia areata, patients with 25%–49% hair loss exhibited higher HADS‐A scores than those with less or more extensive involvement [31]. In psoriasis, discrepancies between symptom severity and perceived burden have been attributed to psychological adaptation or underreporting due to social stigma [32]. These findings suggest that HRQoL impairment is not always aligned with disease severity or duration, since even small affected areas such as ≥ 1% to < 4% or ≥ 4% to < 10% of BSA can have a considerable impact. Substantial psychological distress can occur even in early‐stage or mild cases, emphasizing the need for appropriate support from the earliest phases of the disease.

While this study provides insights into the impact of vitiligo on HRQoL and psychological well‐being, it also has several limitations. First, this study was conducted using a survey panel, and therefore, the generalizability of the results should be interpreted with caution due to possible selection bias. Second, only adult patients aged 18 years or older were included; HRQoL in pediatric patients with vitiligo was not evaluated. Third, disease diagnosis and severity were based on patient self‐reports, in which patients indicated they had been diagnosed by a physician. These diagnoses may not have been made or verified by a dermatologist. As a result, there is a possibility that other causes of depigmentation may have been included. Fourth, 50.2% of the participants reported depigmentation affecting ≤ 1% of BSA, which may further limit the interpretability of disease burden in this cohort. Lastly, we used the SF‐12v2 and DLQI to assess HRQoL. Although the SF‐12v2 has been shown to be equivalent to the longer 36‐Item Short Form Survey [33], the shorter format may increase variability. The DLQI is not specific to vitiligo and includes items on symptoms such as itching and pain that are not typically associated with vitiligo. A vitiligo‐specific HRQoL instrument, the Vitiligo‐Specific Quality‐of‐Life Instrument (VitiQoL), has been developed [34], but a validated Japanese version is not yet available.

In conclusion, this study demonstrated that patients with vitiligo in Japan experience worse HRQoL, particularly in social functioning, and notable symptoms of anxiety and depression. Disease characteristics such as affected BSA, disease duration, and history of relapse were associated with worse HRQoL. These findings underscore the importance of active treatment and sustained disease management, regardless of symptom severity, to support patients' overall well‐being.

## Ethics Statement

The study protocol was reviewed and approved by the RIHDS Ethics Review Committee (Tokyo, Japan; https://rihds.org/ethic/) (approval number: RI2024003) before study initiation.

## Consent

All participants provided informed consent prior to completing the online survey.

## Conflicts of Interest

Naoki Oiso received honoraria for consultancy for this study from Pfizer Japan Inc. Naoki Oiso is an Editorial Board member of The Journal of Dermatology and a co‐author of this article. To minimize bias, he was excluded from all editorial decision‐making related to the acceptance of this article for publication. Yasutaka Mizoro, Kazumasa Kamei, Kenichi Yamanaka, Masato Hoshi, and Kouki Nakamura are employees of Pfizer Japan Inc. Kazumasa Kamei, Masato Hoshi, and Kouki Nakamura hold stocks in Pfizer Inc.

## Supporting information


**Figure S1:** Non‐transformed scores (0–100) of SF‐12v2 subscales. SF‐12v2, 12‐item Short Form Health Survey version 2; PF, physical functioning; RP, role physical; BP, bodily pain; GH, general health; VT, vitality; SF, social functioning; RE, role emotional; MH, mental health.

**Figure S2:** SF‐12v2 component summary scores by disease duration. SF‐12v2, 12‐item Short Form Health Survey version 2; PCS, physical component summary; MCS, mental component summary; RCS, role/social component summary.

**Figure S3:** SF‐12v2 component summary scores by affected BSA. BSA, body surface area; SF‐12v2, 12‐item Short Form Health Survey version 2; PCS, physical component summary; MCS, mental component summary; RCS, role/social component summary.

**Table S1:** Correlation between HRQoL measures.

**Table S2:** Association between disease duration or affected body surface area and HRQoL.

## Data Availability

The data that support the findings of this study are available from the corresponding author upon reasonable request.
