# Supplementary material for: Health‐Related Quality of Life and Psychological Burden of Patients With Vitiligo in Japan
Source: J Dermatol. 2025 Nov 27;53(2):200–9. doi: 10.1111/1346-8138.70059 (PMC12877968; doi:10.1111/1346-8138.70059)
Supplement: Supplementary file 1 — Figure S1: Non‐transformed scores (0–100) of SF‐12v2 subscales. SF‐12v2, 12‐item Short Form Health Survey version 2; PF, physical functioning; RP, role physical; BP, bodily pain; GH, general health; VT, vitality; SF, social functioning; RE, role emotional; MH, mental health. [file JDE-53-200-s001.pdf]

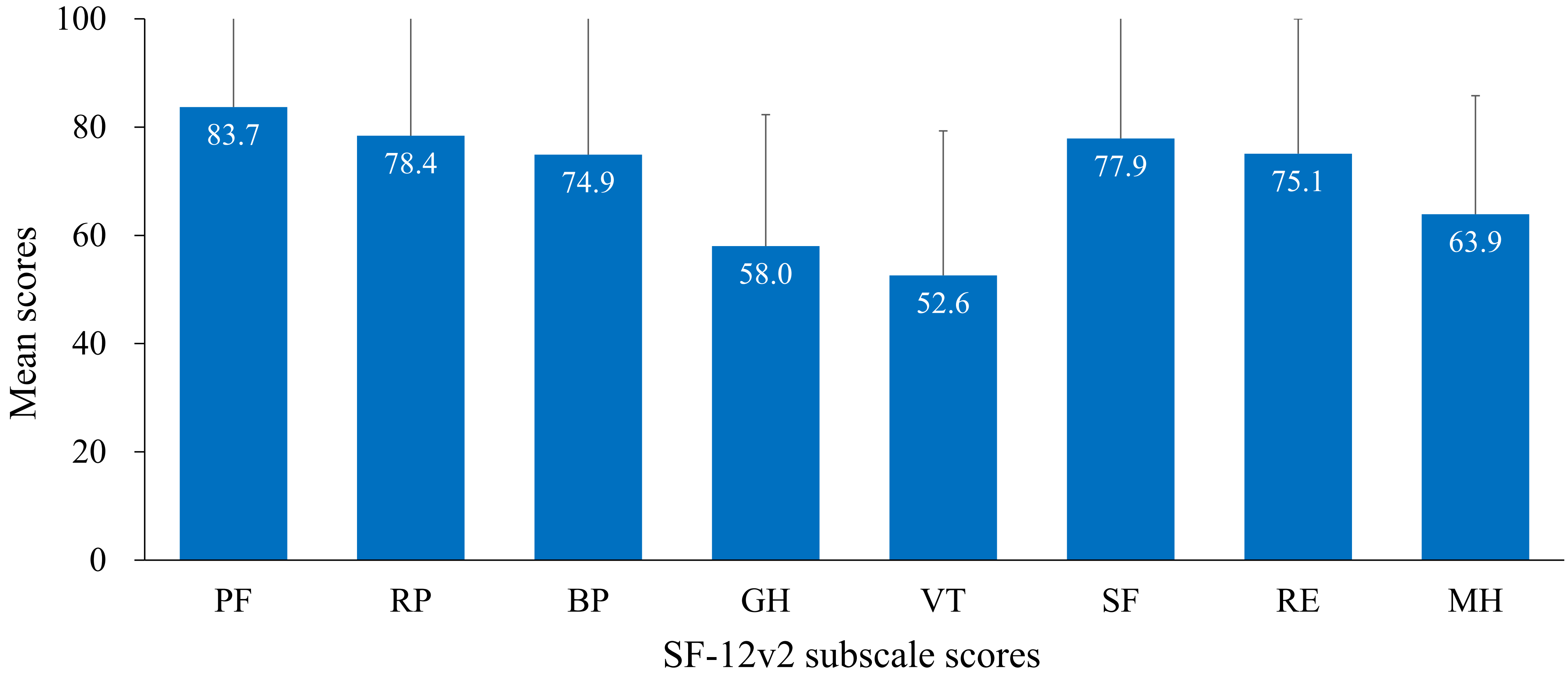

Figure S1. Non-transformed scores (0–100) of SF-12v2 subscales

SF-12v2, 12-item Short Form Health Survey version 2; PF, physical functioning; RP, role physical; BP, bodily pain; GH, general health; VT, vitality; SF, social functioning; RE, role emotional; MH, mental health
