# Supplementary material for: Health‐Related Quality of Life and Psychological Burden of Patients With Vitiligo in Japan
Source: J Dermatol. 2025 Nov 27;53(2):200–9. doi: 10.1111/1346-8138.70059 (PMC12877968; doi:10.1111/1346-8138.70059)
Supplement: Supplementary file 2 — Figure S2: SF‐12v2 component summary scores by disease duration. SF‐12v2, 12‐item Short Form Health Survey version 2; PCS, physical component summary; MCS, mental component summary; RCS, role/social component summary. [file JDE-53-200-s004.pdf]

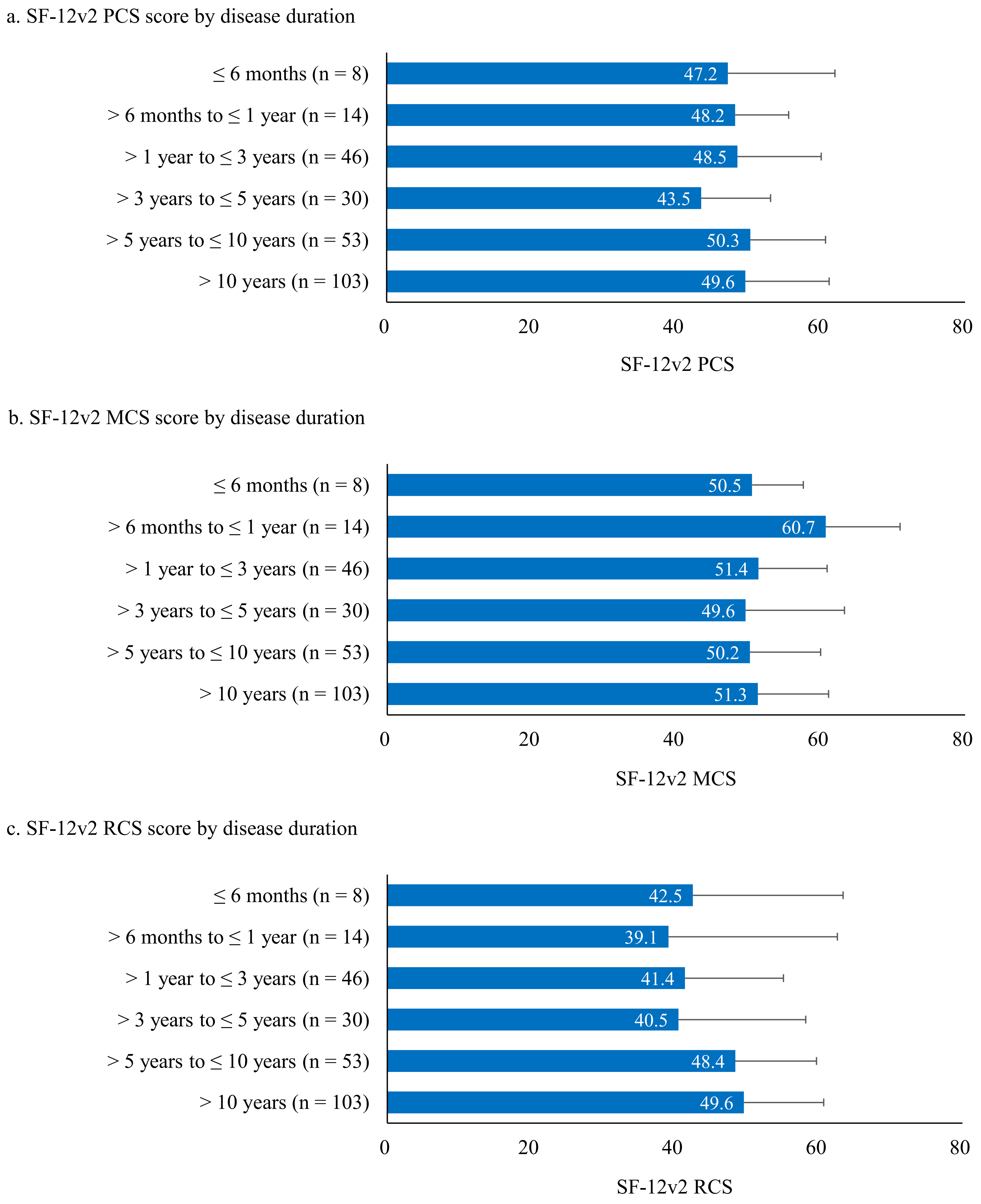

Figure S2. SF-12v2 component summary scores by disease duration

SF-12v2, 12-item Short Form Health Survey version 2; PCS, physical component summary; MCS, mental component summary; RCS, role/social component summary
