# Supplementary material for: Health‐Related Quality of Life and Psychological Burden of Patients With Vitiligo in Japan
Source: J Dermatol. 2025 Nov 27;53(2):200–9. doi: 10.1111/1346-8138.70059 (PMC12877968; doi:10.1111/1346-8138.70059)
Supplement: Supplementary file 4 — Table S1: Correlation between HRQoL measures. [file JDE-53-200-s005.pdf]

Table S1. Correlation between HRQoL measures

		SF-12v2 PCS	SF-12v2 MCS	SF-12v2 RCS	DLQI	HADS-A	HADS-D
SF-12v2 PCS	Correlation coefficient	—					
	R^2	—					
SF-12v2 MCS	Correlation coefficient	−0.016	—				
	R^2	0.000	—				
SF-12v2 RCS	Correlation coefficient	−0.069	**−0.256*****	—			
	R^2	0.005	0.065	—			
DLQI	Correlation coefficient	**−0.321*****	−0.072	**−0.409*****	—		
	R^2	0.103	0.005	0.167	—		
HADS-A	Correlation coefficient	**−0.194****	**−0.300*****	**−0.535*****	**0.605*****	—	
	R^2	0.038	0.090	0.287	0.366	—	
HADS-D	Correlation coefficient	**−0.231*****	**−0.356*****	**−0.449*****	**0.562*****	**0.801*****	—
	R^2	0.053	0.127	0.201	0.315	0.641	—

Spearman's rank correlation test was used to determine the correlation coefficients (ρ) and their p values.
DLQI, Dermatology Life Quality Index; HADS-A, Hospital Anxiety and Depression Scale-anxiety; HADS-D, Hospital Anxiety and Depression Scale-depression; HRQoL, health-related quality of life; MCS, mental component summary; PCS, physical component summary; RCS, role/social component summary; SF-12v2, 12-item Short Form Health Survey version 2
** $p < 0.01$, *** $p < 0.001$
