# Supplementary material for: Health‐Related Quality of Life and Psychological Burden of Patients With Vitiligo in Japan
Source: J Dermatol. 2025 Nov 27;53(2):200–9. doi: 10.1111/1346-8138.70059 (PMC12877968; doi:10.1111/1346-8138.70059)
Supplement: Supplementary file 5 — Table S2: Association between disease duration or affected body surface area and HRQoL. [file JDE-53-200-s003.pdf]

Table S2. Association between disease duration or affected body surface area and HRQoL

| | Disease duration | | Affected body surface area | |
|---|---|---|---|---|
| | Linear contrast estimate | Quadratic contrast estimate | Linear contrast estimate | Quadratic contrast estimate |
| SF-12v2 PCS | 1.569 | 1.914 | −4.185 | −0.258 |
| SF-12v2 MCS | −3.514 | −0.636 | −2.372 | 0.075 |
| SF-12v2 RCS | **7.489**$^{*}$ | 4.901 | −2.276 | **8.143**$^{*}$ |
| DLQI | **−2.927**$^{*}$ | **−4.803**$^{***}$ | 2.686 | **−6.544**$^{***}$ |
| HADS-A | −0.651 | **−2.943**$^{**}$ | 0.141 | **−3.408**$^{**}$ |
| HADS-D | −0.129 | **−3.258**$^{***}$ | 1.489 | −1.479 |

Linear contrast estimate: first-order linear contrast in ANOVA (linear trend). Quadratic contrast estimate: second-order linear contrast in ANOVA (cup-shaped trend). Positive values indicate a cup-shaped trend; negative values indicate a bell-shaped trend.

DLQI, Dermatology Life Quality Index; HADS-A, Hospital Anxiety and Depression Scale-anxiety; HADS-D, Hospital Anxiety and Depression Scale-depression; HRQoL, health-related quality of life; MCS, mental component summary; PCS, physical component summary; RCS, role/social component summary; SF-12v2, 12-item Short Form Health Survey version 2

$^{*}$ $p < 0.05$, $^{**}$ $p < 0.01$, $^{***}$ $p < 0.001$
